# Aberrant Insula-Centered Functional Connectivity in Psychogenic Erectile Dysfunction Patients: A Resting-State fMRI Study

**DOI:** 10.3389/fnhum.2017.00221

**Published:** 2017-05-16

**Authors:** Yue Wang, Minghao Dong, Min Guan, Jia Wu, Zhen He, Zhi Zou, Xin Chen, Dapeng Shi, Jimin Liang, Xiangsheng Zhang

**Affiliations:** ^1^Engineering Research Center of Molecular and Neuro Imaging of Ministry of Education, School of Life Science and Technology, Xidian UniversityXi’an, China; ^2^Henan Andrological Academician Workstation of Basic and Clinical Research, Henan Provincial People’s HospitalZhengzhou, China; ^3^Department of Interventional Radiology, Henan Provincial People’s HospitalZhengzhou, China; ^4^School of Foreign Languages, Northwestern Polytechnical UniversityXi’an, China; ^5^Department of Radiology, Henan Provincial People’s HospitalZhengzhou, China; ^6^Department of Uro-Andrology, Henan Provincial People’s HospitalZhengzhou, China

**Keywords:** psychogenic erectile dysfunction, insula-centered, functional connectivity, resting state, functional magnetic resonance imaging

## Abstract

Most previous studies exploring the neural mechanism of psychogenic erectile dysfunction (pED) focused on brain activity under tasks. We suggest that the resting brain activity is equally important in pED studies, in that the patterns of spontaneous neural activities is independent of modalities of sensory input, therefore providing substantial information regarding the central mechanism of pED. Our previous study reported the altered baseline activity in right anterior insula (aINS) in pED patients. Also, the insula is a pivotal region in sexual behavior, which is suggested to be able to directly mediate erection. Therefore, the current study employed resting-state fMRI to examine alterations in functional connectivity (FC) of the aINS comparing pED patients with matched control subjects. After rigorous participant inclusion procedure, 27 pED patients and 27 healthy male controls were enrolled. Our results elucidated the disrupted homogeneity within the right aINS and aberrant connection patterns between the right aINS and the right dorsolateral prefrontal cortex (dlPFC), as well as the right aINS and the right temporoparietal junction (TPJ) respectively in pED group, as compared with the healthy controls. In conclusion, our results demonstrated the aberrant insula-centered FC in pED, which may be related to the abnormal representation of internal bodily state or needs in pED patients and thus further affect the inhibitory control in the sexual context. We hope that these findings may shed new light on the understanding of the central mechanism of pED.

## Introduction

Erectile dysfunction (ED) is defined as the disability to gain or maintain adequate penile erection during sexual intercourses (Wespes et al., 2002). Psychogenic ED (pED), a subtype of ED, is due predominantly or exclusively to psychological or interpersonal factors (Rosen, 2001). PED patients consist of 90% of ED population under the age of 40 (Rosen, 2001; Lewis et al., 2004), but its neural mechanism has not been sufficiently clarified.

In recent years, neuroimaging studies investigating the neural mechanism underlying pED have observed both structural and functional alterations in multiple cortical and subcortical brain regions, such as the insula, the supramarginal, the superior/inferior parietal lobes, the (ventral) medial prefrontal cortex, the anterior/posterior cingulate cortex, the hippocampus, the nucleus accumbens, the hypothalamus and others (Cera et al., 2012a,b, 2014; Zhao et al., 2015a,b). More importantly, the insula, the medial/dorsolateral prefrontal cortex and the inferior parietal cortex are found to directly correlate with male erection in the context of sexual relevant stimulus (Arnow et al., 2002; Ferretti et al., 2005; Moulier et al., 2006; Cera et al., 2012b). These findings indicated that constellation of brain regions are involved in sexual stimuli processing.

Our recent study (Guan et al., 2016) reported changed baseline brain activity of the insula, especially the right aINS, in pED patients, whose level of baseline activity was correlated with erection scores. The insula has been proven to be pivotal in the mediation of sexual behavior. Human studies have identified the involvement of insula in response to sexual stimulus (Craig, 2002; Ortigue et al., 2007), especially its direct role in modulating penile erection (Arnow et al., 2002; Ferretti et al., 2005; Cera et al., 2012b). In animal studies, the direct participation of insula under the erectile or sexual stimulus have also been observed. Specifically, the insula was activated during the intercourses in rodents (Georgiadis et al., 2012), and it is also activated by sexually arousing stimulus in primates (Ferris et al., 2004). On the other hand, the malfunction of insula is closely related to pED. Studies from other groups (Cera et al., 2014) and our group (Zhao et al., 2015a,b) on the neural accounts of pED patients have explicated the abnormality of the right insula. Therefore, the insula’s involvement in both normal and abnormal sexual behavior, we suggest that the insula is of particular interest to pED research. Moreover, the baseline issue is rudimentary in fMRI studies, particularly for studies adopting tasks, in that the changes in baseline brain activity may smear the spatial activation under task (Di et al., 2013) and bring biased results (Dong et al., 2015). Therefore, in the current study, the region whose baseline changed, i.e., the right aINS, was used as the entry point.

Previous fMRI studies used task paradigms to probe the central mechanism of pED (Cera et al., 2012b, 2014), which are exclusively dependent on visual stimulation. However, sexual response derives from all sensory input modalities (Ferris et al., 2001; Ferretti et al., 2005), as well as imaginary (Rauch et al., 1999). Also, clinical data shows the failure in sexual intercourse is not limited to visual stimulation in pED patients. In other words, the failure in sexual intercourse is independent of sensory input modalities, which suggests that the processing of sexual stimuli may be disrupted. The resting state fMRI (rs-fMRI) investigated the spontaneous neural activities and activities in the absence of tasks. It is useful to examine the neural mechanism of a wide range of social or cognitive brain disorders, such as Alzheimer’s disease (Wang et al., 2007), amnestic mild cognitive impairment (Han et al., 2011), major depressive disorder (Hamilton et al., 2011) and others. We propose that the rs-fMRI is essential. Therefore, the current study used rs-fMRI.

Given the aforementioned facts that: (1) the sexual response, regardless of being typical or atypical, is mediated by spread brain regions rather than a single region; (2) the right aINS’s served a pivotal role in pED; (3) rs-fMRI may provide more fundamental information regarding the central mechanism underlying both normal and abnormal sexual response, in the current study, we employed rs-fMRI and investigated the insula-centered functional connectivity (FC) in pED patients using the FC analysis. The FC analysis describes the pattern of dependence between insula and other brain regions and has been widely used in functional disorder studies (Greicius et al., 2007; Wang et al., 2007; Luo et al., 2011). We suggest that our study provides a novel perspective to understand the neural mechanism of pED. The findings of the current study may help develop new ideas for the diagnosis, treatment and rehabilitation of pED.

## Materials and Methods

### Ethics Statement

The study was approved by the ethics committee of Henan Provincial People’s Hospital and was conducted in accordance with the Helsinki Declaration. Subject’s personal information and their intimacy were protected. The detailed study design was explained to all the subjects and written informed consent was obtained from all the subjects participated.

### Subjects

Fifty-three right-handed male heterosexual pED patients and 34 right-handed male heterosexual normal controls (NC) were recruited. Both groups were managed by Henan Provincial People’s Hospital. The patients were from the in patients department of urology of  Henan Provincial People’s Hospital and the NC group were volunteers recruited through advertisement. All these subjects went through rigorous screening process (e.g., age, handedness, sexual orientation, sexual relations, medical history etc.) before the MRI scanning. Eventually, 27 pED patients (26.58 ± 4.89 years, mean ± SD) and 27 NC (28.39 ± 3.53 years, mean ± SD) were enrolled.

The diagnosis of pED followed the standard clinical guidelines (Wespes et al., 2006): (1) physical examinations, including penile duplex Doppler ultrasonography, RigiScan test and electrocardiogram examination were adopted to insure the absence of vasculogenic, neurogenic, anatomical and drug-induced ED; and (2) neuropsychiatric tests were administered to ensure that none of the participants enrolled in this study met the Diagnostic and Statistical Manual of Mental Disorders, Fifth Edition (DSM-V). Moreover, history of the sexual relations, medical, medication and surgical disorders of each participant have been recorded in detail. The same examinations were performed on the NC group as well.

The exclusion criteria of the subjects were as follows: (1) patients with insufficient duration of ED (less than 6 months); (2) subjects with vascular or genital impairments; (3) subjects diagnosed to have mental disorders; and (4) patients whose medication history contains the drugs without defined pharmacokinetic function or literature to define their effect over the central nervous system. According to the record, patients included in this study have taken tadalafil, sildenafil, vardenafil and apomorphine, and these drugs were easy to be metabolized and causes no permanent effects on the subjects’ central nervous system (Altwein and Keuler, 2001; Porst et al., 2001; Gaines, 2004; Forgue et al., 2006). Subjects were asked to avoid taking these medicines 1 week ahead of MRI data acquisition to ensure they are free of the drug’s impact.

Participants in this study also completed several questionnaires. The International Index of Erectile Function (IIEF; Rosen et al., 1997), a multi-dimensional self-report instrument for clinical evaluation of male sexual function, was used to assess the erectile functioning for all the participants. It is used as the primary endpoint for clinical trials of ED and as a diagnostic evaluation of ED severity (Rosen et al., 2002). Furthermore, Self-Rating Anxiety Scale (SAS; Jegede, 1977) and Self-Rating Depression Scale (SDS; Zung, 1965) were used to evaluate the anxiety and depression levels for all participants involved.

### MRI Data Acquisition

Magnetic resonance imaging (MRI) data in this study was obtained on a 3T GE 750 Discovery MRI scanner (General Electric, Milwaukee, WI, USA), with a dedicated 8-channel head coil. Resting-state functional images were collected using an echo-planar imaging sequence with the following parameters: scan duration = 8 min, repetition time = 2000 ms, echo time = 30 ms, field of view = 24, flip angle = 90°, number of slices = 240, slice thickness = 4 mm, gap = 0 mm, and matrix = 64 × 64. During the whole scanning process, the subjects were asked to keep their eyes closed, their head still and stay awake. After the scanning session, the subjects were asked whether they had fallen asleep during the scanning.

### Data Analysis

#### MRI Data Preprocessing

Preprocessing of functional scans were conducted using the dpabi toolbox v2.0^1^, including: (1) while discarding the first 10 volumes to avoid non-equilibrium effects of magnetization and allow the participants to accommodate to the EPI scanning environment; (2) slice timing correction to correct for the acquisition delay between slices; (3) realignment to match each functional volume to the reference volume, the estimated translation and rotation parameters for each volume in the time course of each subject were not larger than 2 mm and 2°; (4) regressing the effects of head motion parameters (using the rigid-body 6-parameter model), white matter and cerebro-spinal fluid signals; (5) spatial normalization and resampled the functional images to 3 mm isotropic voxels; (6) spatially smoothen the functional images with an isotropic Gaussian kernel (FWHM = 6 mm); (7) remove the linear trend; and (8) temporal filtering (0.01–0.08 Hz; Biswal et al., 1995; Lowe et al., 1998) to reduce the effect of low-frequency drifts and high-frequency noise. The voxel size was [3 3 3] after preprocessing.

#### Seed Region Definition

**Figure 1:**
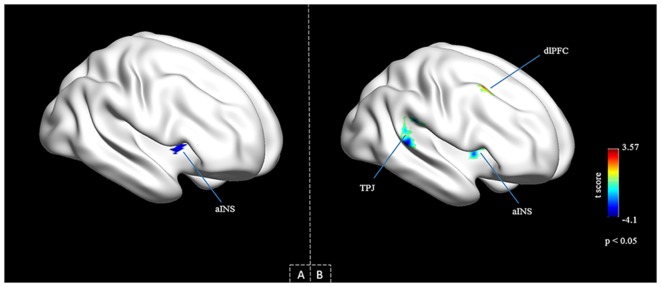
**Group comparison of the insula-centered functional connectivity (FC). (A)** Seed region in the right aINS with altered baseline activities in psychogenic erectile dysfunction (pED).** (B)** Cortical areas that show significant differences between the two groups (pED vs. normal controls (NCs)). Aberrant connectivity patterns were observed within the right aINS, and between the right aINS and the right dlPFC and the right temporoparietal junction (TPJ) respectively. Between group differences are assessed by means of two-sample *t* analysis (*p* < 0.05 AlphaSim corrected). Abbreviations: aINS, anterior insula, dlPFC, dorsolateral prefrontal cortex, TPJ, the temporoparietal junction.

With right aINS being the main outcome of previous study reporting the baseline alteration in pED patients (results of the two-sample *t* analysis, i.e., the pED patients vs. the healthy control; Guan et al., 2016), the current study used the same group of subject as that used previously and utilized this area to be the ROI (cluster size = 40), which was found previously to correlated with the erectile function. Confirmation of the ROI to be part of the right aINS was conducted using the clustering method and template proposed by Kelly et al. (2012; Figure 1A), and the mask used in current study consisted of the voxels exceeding the statistical threshold in the two-sample *t* analysis (*p* < 0.05, FWE corrected).

#### Functional Connectivity Analysis

FC calculation was applied to the preprocessed fMRI data for measurement of temporal synchronization between seed region and each voxel across the whole brain. Individual z value maps were obtained with a Fisher’s z transformation imposed on the FC outcomes to make the data in normal distribution. FC-based comparisons of insula-centered FC performance were performed between groups of pED and NC using two-sample *t* tests. The results were considered significant when exceeding the threshold of *p* < 0.05 (AlphaSim corrected). The AlphaSim correction in this study was performed using the REST toolbox v1.8^2^ and the voxel level and cluster level *p* thresholds were set to 0.005 and 0.05 respectively. The spatial smoothness was estimated based on the FSL script easythresh^3^.

#### Correlation Analysis

To examine the relationship between the behavioral/psychological measurement and the brain measurement, voxel-wise correlation analysis was performed between IIEF, SAS, SDS and FC outcomes. The results were considered significant above a threshold of *p* < 0.05 (AlphaSim corrected). The AlphaSim correction in this study was performed using the REST toolbox v1.8^4^ and the voxel level and cluster level *p* thresholds were set to 0.005 and 0.05 respectively.

In addition, correlation between IIEF and SAS/SDS were calculated.

#### *Post hoc* Analysis

In the present samples, both of the SAS and SDS scores showed significant differences between groups. Also, the male sexual functioning (measured by IIEF scale) was found to be negatively correlated with the SAS scores and SDS scores respectively (Pearson’s *r* = −0.3787, *p* < 0.01 and Pearson’s *r* = −0.5407, *p* < 0.001, respectively). Therefore, we repeated the between-group comparison in FC analysis after controlling SAS and SDS scores to ensure that these variables were not driving our results.

## Results

### Demographic and Psychological Data

Demographic and psychological features of pED and NC groups are summarized in Table 1. The two groups were not significantly different in age and level of education (*p* > 0.05, two-sample *t* test, two-tailed). The pED patients had a lower IIEF score (*p* < 0.05, two-sample *t* test, two-tailed), higher SAS (*p* < 0.05, two-sample *t* test, two-tailed) and SDS scores (*p* < 0.005, two-sample *t* test, two-tailed) than NC.

**Table 1:** **Demographic data and clinical variables of psychogenic erectile dysfunction (pED) patients and normal controls (NCs) (pED vs. NC)**.

	pED (M ± SD)	NC (M ± SD)	*p* value
**Demographics**			
Age (years)	26.58 ± 4.89	28.39 ± 3.53	0.148
Education (years)	11.52 ± 2.87	13.37 ± 1.78	0.34
**Psychological data**			
IIEF-total	31.38 ± 12.14	64.71 ± 5.49	<0.001
SAS	45.31 ± 7.01	39.71 ± 9.01	0.021
SDS	49.81 ± 9.53	39.38 ± 9.06	<0.001

*Abbreviations: pED, psychogenic erectile dysfunction; NC, normal controls; M, mean; SD, Standard Deviation; IIEF, International Index of Erectile Function; SAS, Self-Rating Anxiety Scale; SDS, Self-Rating Depression Scale. The p-value was obtained with a two-tailed two-sample t tests*.

None of the subjects (SAS < 60, SDS < 70) reached severe anxiety and depression levels (Zung, 1965, 1971).

### Between-Group FC Results

The seed region in the right aINS that showed altered baseline activities as revealed in previous study of our group using amplitude of low frequency fluctuation is demonstrated in Figure 1A (Guan et al., 2016). The area was defined as right aINS using clustering method proposed by Kelly et al. (2012).

When part of the right aINS was used as seed in FC analysis in rs-fMRI, significant group-wise temporal synchronism differences with the ROI were observed in aINS, dlPFC and temporoparietal junction (TPJ) in the right hemisphere of brain (AlphaSim correction, the corresponding statistical level is set at |*t*| > 2.932 (*p* < 0.005 at individual voxel level) and cluster size > 45 voxels (search volume = 67541 voxels)). The pED group showed significant lower *t* values in aINS (48 3 0; *t* = −4.4688) and TPJ (54 −30 21; *t* = −4.1016), while significant higher *t* values in dlPFC (36 18 51; *t* = 3.5660) was observed in this group, which corresponds to a corrected *p* < 0.05 (Table 2, Figure 1B). Note that the aINS identified in this analysis is not spatially overlapped with the ROI of this study.

**Table 2:** **Brain areas revealed by two-sample *t* tests on zFC maps (pED vs. NC, *p* < 0.005)**.

Cluster	Size	Side	BA	*x*	*y*	*z*	*t* value	*p* value
Anterior insula	63	R	48	48	3	0	−4.4688	0.0038
The temporoparietal junction	86	R	22/42/48	54	−30	21	−4.1016	0.0027
Dorsolateral prefrontal cortex	47	R	8/9	36	18	51	3.5660	0.0036

*Brain region are listed with MNI coordinates of the peaks of the cluster and the corresponding t and p values. Abbreviations: BA, Brodmann’s area; R, right*.

### Results of the Correlation Analysis

**Figure 2:**
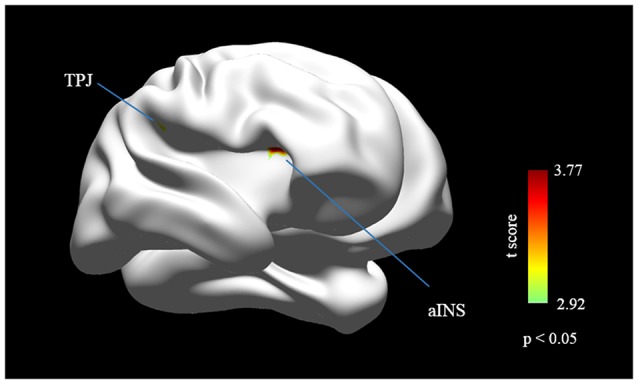
**Cortical areas that show significant correlation between the international index of erectile function (IIEF) score and the FC outcomes**. The IIEF score is positively correlated with the FC in right aINS and right TPJ (*p* < 0.05, AlphaSim corrected). Abbreviations: aINS, anterior insula; dlPFC, dorsolateral prefrontal cortex; TPJ, the temporoparietal junction.

Correlation analysis was applied between the FC calculation outcomes and the behavioral/psychological data (AlphaSim correction, the corresponding statistical level is set to |*t*| > 2.932 (*p* < 0.005 at individual voxel level) and cluster size > 10 voxels (search volume = 67541 voxels)), which corresponds to a corrected *p* < 0.05. Significant positive correlations were observed between IIEF and aINS (36 15 12; *t* = 3.7681) and right TPJ (57 −30 21; *t* = 3.1855; Figure 2, Table 3). None of the significant correlations was observed between zFC results and SAS/SDS. None of the overlapped significant area was observed between the group-wise FC difference map and correlation analysis results between FC values and SAS, SDS scores. When the threshold was slightly liberated, the dlPFC was marginally correlated with IIEF (*p* < 0.01 at individual voxel level). This indicates that the dlPFC was also likely to be closely involved in pED. But, this finding is not the primary finding in the current study.

**Table 3:** **Brain areas revealed by correlation analysis between zFC maps and IIEF scores (pED vs. NC, *p* < 0.005)**.

Cluster	Size	Side	BA	*x*	*y*	*z*	*t* value	*p* value
Anterior insula	27	R	48	36	15	12	3.7681	0.0017
The temporoparietal junction	20	R	48	57	−30	21	3.1855	0.0025

*Brain region are listed with MNI coordinates of the peaks of the cluster and the corresponding t and p values. Abbreviations: BA, Brodmann’s area; R, right*.

### Results of *Post hoc* Analysis

When computing FC analysis, by adding SAS and SDS scores as control variables, the results were not changed. Note that the major findings of the current study were outcomes without SAS and SDS as covariates, in that: (a) evidence showed that impaired sexual function in men is significantly associated with negative psychological effects such as anxiety and depression (Feldman et al., 1994; Sugimori et al., 2005); and (b) we propose that slight anxiety/depression and poor erectile functioning are overall effects of pED and inseparable in pED study.

## Discussion

The current study is the first study investigating the brain FC abnormality in the pED patients using the rs-fMRI. Specifically, in the present study, we investigated the insula-centered FC in pED patients. Aberrant connection patterns were observed within the right aINS, and between the right aINS the right dlPFC and the right TPJ (supramarginal/superior temporal gyrus) respectively, as compared with the NC. For the behavioral data, the pED group showed significant lower erectile functioning scores (*p* < 0.05), higher anxiety scores (*p* < 0.05) and higher depression scores (*p* < 0.005) than NC group, as measured by IIEF, SAS and SDS, respectively. Importantly, the erectile functioning, as indexed by IIEF was significantly correlated with the right aINS and the right TPJ, and had a tendency of anti-correlation with the right dlPFC. Previous fMRI studies on central mechanism of pED patients were initiated to locate single or isolated brain regions that malfunction in response to sexual stimuli (Cera et al., 2012a,b; Zhao et al., 2015a,b). However, the human sexual response is mediated by a constellation of brain regions (Ferretti et al., 2005; Moulier et al., 2006). Therefore, in this study, we investigated the central mechanism of pED through alterations of local FC in pED patients.

The participation of insula in male sexual behavior have been supported by multiple functional neuroimaging studies (Stoléru et al., 1999; Kuhn and Gallinat, 2011), especially its direct role in mediation of penile erection, such as the perceptual processing of penile inputs (Moulier et al., 2006), onset of erection (Cera et al., 2012b), recognition of erection (Arnow et al., 2002) and sustain penile response to erotic stimuli (Ferretti et al., 2005). Moreover, in animal studies, such as rodents and primates, insula was activated in sexual relevant processes, such as copulation (Ferris et al., 2004; Georgiadis et al., 2012). Particularly, recent study have revealed the vital role of insula in the development of pED (Cera et al., 2014), indicating that insula is important in sexual relevant processes, which can also be used as an important entry point for pED study. In the current study, compared with healthy NC group, the inter-region functional homogeneity is disrupted in the right aINS, as revealed by altered FC within the right insula in the pED group. This result is consistent with previous findings using task paradigm. In detail, Cera et al. (2014) observed a decreased level of the FC in correspondence of the insula in pED patients and implied an altered inter-region function of insula in pED patients. Specifically, the aINS participates in interoceptive awareness (Craig, 2002), social emotional (Immordino-Yang et al., 2009) and cognitive processes (Craig, 2009). Numerous studies have proved the key role played by right aINS in awareness of sexual arousal (SA) and sexual urges (Arnow et al., 2002; Ferretti et al., 2005; Moulier et al., 2006; Mouras et al., 2008). The involvement of this region in pED is also supported by its correlation with erectile function (*p* < 0.05, multiple-corrected), indexed by IIEF, indicating the direct linkage between right aINS and sexual relevant internal bodily state and/or needs representations in pED patients. According to the fact that emotional states are integrated with interoceptive states in the representation of the subjective feelings of the moment (Craig, 2009), the disruption of insula functioning may mainly reflect the abnormal representation of internal bodily state and/or needs of pED patients in sex-relevant conditions.

The right dlPFC showed significantly higher connection strength with the right aINS in pED group compared with NC. Joint activations of dlPFC and insula have been observed in multiple sexual stimulus paradigms (Ferretti et al., 2005), indicating the vital role of FC pattern between these two regions in sex-related processes. Moreover, anatomically, the right dlPFC was connected to the right aINS through white matter (Flynn et al., 1999). Lines of evidence have shown that dlPFC was actively engaged in the processing of sex relevant information. Specifically, lesion and neuroimaging studies suggest that dlPFC was important in regulating the processing of sexual information, especially its specific role in order to guide the inhibition or elicitation of sexual response (Terzian and Ore, 1955; Freeman, 1973). In this study, we found the tendency of negative correlation between insula-centered FC values and erectile functioning index in right dlPFC, as measured by IIEF, and propose the adverse effect of dlPFC in sexual information processing. Its role in pED is further supported by previous sex-related studies, where the inhibitory role of dlPFC were investigated by both forward and reverse experiments. Specifically on one hand sexual inhibition in patients with prefrontal lesions was observed in previous studies (Kreutzer and Zasler, 1989; Zasler, 1997), which have been interpreted by the failed recruitment of dlPFC in resolving difficult personal moral dilemmas and conflictive decision (Leon-Carrion et al., 2007). Similarly, in healthy males, greater activation in dlPFC was also observed during viewing of sexually explicit scenes when no extra constraint was imposed on them (Anderson et al., 1999; Greene et al., 2004). On the other hand, the enhanced sexual inhibitory was found to be associated with the dlPFC activity in a volitional inhibitory paradigm, the activation of right dlPFC was observed to positively correlate with the decreased intensity of the SA induced by the erotic film excerpts (Beauregard et al., 2001). Therefore, we propose that the altered FC between the right dlPFC and the right aINS in this study may be associated with the abnormal inhibitory control of sexual response in pED patients. The psychological or interpersonal pathogenic factors in the development of this disorder may have worked as a repeated volitional inhibitory training, and this kind of inhibition of sexual response may act on the attentional processing of external sexual stimuli and this attention-related conjecture is consistent with previous study by Menon and Uddin (2010), then lead to excessive inhibition of the subject’s sexual needs and resulted in the experience-dependent brain plasticity alteration in pED patients.

Compared with the NC group, decreased insula-centered FC in pED patients was observed in region of right TPJ. Structural connections between the TPJ and insula have previously been identified in the primate (Pandya and Kuypers, 1969) and human (Saur et al., 2008; Umarova et al., 2010) brains. Further, TPJ and insula have been observed to co-activate under sexual context (Ortigue et al., 2007; Woodard et al., 2013). The right TPJ is a key component of a right-lateralized ventral attention network (Corbetta et al., 2008), which is involved in the saliency-based attention reorienting (Downar et al., 2002; Lepsien and Pollmann, 2002; Indovina and Macaluso, 2007). In this study, we reported correlation between insula-centered FC values in right TPJ and erectile functioning indexed by IIEF, indicating the involvement of the FC pattern between right TPJ and insula in sex-related processes. In former studies, right TPJ have been observed to exhibit stronger activation during exposure to sexual stimuli and the activation of which was associated with the attention intensity to targets that were perceived as sexual cues (Stoléru et al., 2012; Seok and Sohn, 2015). Furthermore, right TPJ was found to participate in the mediation of the motivational component of SA (Zhao et al., 2015b). In one study on problematic hypersexual behavior (PHB), greater attention has been observed in TPJ to be positively associated with higher levels of sexual desire in PHB patients (Seok and Sohn, 2015). It seems that the FC alteration between right aINS and TPJ may indicate a lower level of sexual desire of pED patients (in other words, the insufficient motivation component of SA), which is likely to be attributed to the insula’s inaccurate representation of internal bodily needs and the following insufficient concentration on the sexual targets. However, due to the limited explanatory power of data in this study, this conjecture needs to be verified in further experiments.

Several limitations should be taken into consideration when interpreting the findings in this study. First, as for all cross-sectional studies, confounding factors, such as the interpersonal factors or pathogenic factors, cannot be ruled out as possible explanations for the observed differences between groups. Although it is not likely the case we should still be discreet to claim that the observed FC alterations are actually caused by pED, or simply be the result of a rare phenotype within the general population. Longitudinal experiments should be considered in further studies to further test the hypothesis. Second, sample size in this study is comparatively small, larger samples are encouraged to repeat the current findings. Our on-going study is recruiting more subjects and would help to settle the issue. Thirdly, this study did limit the inclusion criterion to drug-naïve subjects patients. This challenge is the same for all other studies involving subjects. The pED group in this study were all in-patients subjects and drug naïve pED patients was pretty hard to find in the in-patients department; and, the cases are especially rare for patients with history longer than 6 months. But, we did undertake rigorous measures to ensure the drug effect on the brains had been metabolized before scanning (as explicated in the “Subject” Section).

## Conclusion

The current study, for the first time, investigated the FC abnormality in the pED patients using the rs-fMRI. Our data suggest the presence of aberrant insula-centered FC underlying pED, which may indicate an abnormal representation of internal bodily states/needs and excessive inhibition control during sex-related conditions in pED patients. We consider our findings to be helpful in providing new perspectives in the treatment of pED.

## Author Contributions

MD, MG, XZ: designed the experiments. MD, MG, DS, ZZ: performed the experiments. YW: analyzed the data and wrote the article. ZH: Re-Run the data and checked all the technical details. MD, XC, JL: contributed reagents/materials/analysis tools. JW, JL: contributed to the writing of the manuscript.

## Conflict of Interest Statement

The authors declare that the research was conducted in the absence of any commercial or financial relationships that could be construed as a potential conflict of interest.

## Footnotes

^1^http://rfmri.org/dpabi

^2^http://restfmri.net

^3^https://fsl.fmrib.ox.ac.uk

^4^http://restfmri.net
